# The learning curve on robot-assisted transaxillary thyroidectomy performed by a single endocrine surgeon in a third-level institution in Europe: a cumulative sum (CUSUM) analysis

**DOI:** 10.1007/s13304-023-01619-z

**Published:** 2023-08-02

**Authors:** Gabriele Materazzi, Piermarco Papini, Lorenzo Fregoli, Riccardo Morganti, Andrea De Palma, Carlo Enrico Ambrosini, Leonardo Rossi

**Affiliations:** 1 Endocrine Surgery Unit, Department of Surgical, Medical and Molecular Pathology and Critical Area, University of Pisa, Pisa, Italy; 2 Section of Statistics, University of Pisa, Pisa, Italy

**Keywords:** Robot-assisted transaxillary thyroidectomy, Robotic surgery, Thyroid, Learning curve, Remote access, CUSUM analysis

## Abstract

Robot-assisted transaxillary thyroidectomy is widely performed in Asian countries, although it is still under discussion in the Western World. However, there have been few studies reporting on the learning curve of robot-assisted transaxillary thyroidectomy. We used the cumulative sum (CUSUM) analysis to assess the learning curve of gasless robot-assisted transaxillary thyroidectomy at a third-level institution in Europe. We included all consecutive patients operated by a single surgeon without previous experience of robotic surgery from February 2012 to January 2023. The primary endpoint of the study was the learning curve extracted from the median operative time using the CUSUM method for the quantitative assessment. Overall, 583 patients were enrolled. The median operative time for thyroid lobectomy and total thyroidectomy was 70 and 90 min, respectively. The CUSUM analysis showed that the learning curve for thyroid lobectomy and total thyroidectomy is 66 and 56 cases, respectively. Moreover, the presence of thyroiditis resulted associated with shorter operative time for total thyroidectomy (*p* = 0.044), whereas no factors resulted associated with surgical complications. The learning curve for performing robotic transaxillary thyroid lobectomy for a surgeon without previous robotic experience is 66 cases. After that, 56 cases must be performed to acquire proficiency in robotic transaxillary total thyroidectomy. Training programs may reduce the slope of the learning curve.

## Introduction

Conventional open thyroidectomy is a safe technique but leaves an anterior neck scar that often can be distressing for patients, in particular for young women who represent the major part of candidates to this procedure [1]. Therefore, minimally invasive and remote-access techniques have been developed to provide better cosmetic outcomes [2–8].

Robot-assisted transaxillary thyroidectomy (RATT) using the da Vinci robotic system (Intuitive Surgical Inc., Sunnyvale, CA, U.S.) was first developed by Chung and Colleagues in Korea in 2007 [9, 10]. The da Vinci robotic system (Intuitive Surgical Inc., Sunnyvale, CA, U.S.) has been reported to provide excellent 3-D visualization, magnified view, tremor-filtering technology, and large movement flexibility due to the multi-articulated robotic arms with 7 degrees of freedom [11, 12].

This approach gained widespread acceptance especially in Asian countries, whereas its success in Western World came more slowly [13]. This aspect was primarily related to issues concerning reimbursements, insurance, and medicolegal litigations, as well as to differences in terms of anthropometric characteristics, thyroid disease features, and cultural issues. In addition to these constraints, the manufacturer of the robotic system has also limited its support for thyroid resection, which further complicates the adoption process and potential integration of RATT in surgical practices [14].

Moreover, RATT requires surgeons to become familiar with the lateral view proper of the approach, the robotic instruments’ handling, and the lack of haptic feedback. Nowadays, a few studies investigated the learning curve of this technique, in particular considering Western World patients [15–17].

We aim to assess the learning curve of a surgeon with a huge experience in endocrine surgery, but without previous experience in robotic surgery, in performing RATT at a third-level institution in Europe.

## Materials and methods

We conducted a retrospective study using a prospectively maintained database for data collection and analysis. All consecutive patients operated by means of RATT by a single surgeon (GM) at the Endocrine Surgery Unit, University Hospital of Pisa, from February 2012 to January 2023 were enrolled. The surgeon was experienced in the conventional open thyroidectomy and minimally invasive video-assisted thyroidectomy (MIVAT) [18], but had no experience in robotic surgery.

All patients provided informed consent and received an extensive explanation of the robotic procedure and were counseled about the conduct, risks and alternatives of RATT and conventional thyroidectomy.

All procedures were performed using the da Vinci robotic system (Intuitive Surgical Inc., Sunnyvale, CA, U.S.), either the SI or the XI versions, using a single axillary incision and three robotic arms. Data regarding patients’ demographics, thyroid volume, diameter of the nodules, extent of surgery, operative time, postoperative length of hospital stay, postoperative surgical complications, and histology were recorded. All patients were preoperatively evaluated by means of neck ultrasound, ultrasound-guided fine-needle aspiration cytology if required, and blood test to assess the thyroid function.

The choice of surgery between thyroid lobectomy (TL) and total thyroidectomy (TT) was in accordance with the American Thyroid Association management guidelines for adult patients with thyroid nodules and differentiated thyroid cancer at the time [19, 20].

Operative time was defined from the skin incision to the skin closure. Postoperative hypocalcemia was defined as an albumin-corrected calcium level of < 8.0 mg/dl or inability to interrupt calcium therapy [21]. Recurrent laryngeal nerve (RLN) palsy was diagnosed by an independent otolaryngologist in case of documented vocal cord mobility alteration at fiberoptic laryngoscopy. We used the cutoff of 6 months to discriminate between transient and permanent postoperative hypocalcemia and RLN palsy. Bleeding was defined as a hemorrhage that could be treated by surgical revision or by conservative management (cervical hematoma).

The primary endpoint of the study was the learning curve extracted from the median operative time; secondary endpoints of the study were the risk factors associated with surgical complications and prolonged operative time.

### Statistical analysis

The cumulative sum (CUSUM) method is a sequential analysis technique used for the quantitative assessment of the learning curve [15, 22]. The CUSUM analysis provides the estimation of the cumulative differences between the observed data and the target values.

The trend of the learning curve was indicated by the slope of the CUSUM graph that fits a polynomial curve. A positive slope implied that the target was not achieved, whereas a negative slope suggested that the target was exceeded. The point where the slope changed from positive to negative reflected that the learning curve was overcame. We performed the CUSUM analysis taking into consideration the median operative time.

Categorical data were described with the absolute and relative (%) frequency, whereas continuous data were summarized with the mean value and standard deviation.

A simple linear regression between “operative time” and “number of cases” stratified for the extent of surgery (TL and TT) was performed. To compare categorical and continuous factors influencing the outcome variables as operative time (dichotomized either for TL and TT) and postoperative complications, Chi-square test and *t *test for independent samples (two-tailed) were applied, respectively.

The significance was set at 0.05, and all analyses were carried out employing Microsoft Excel program and the SPSS v.28 technology (IBM Corp., Armonk, NY, USA).

## Results

The clinicopathological features and surgical outcomes of patients are summarized in Table 1. The study population included 583 procedures: 363 (62.3%) TL and 220 (37.7%) TT. The patient’s mean age was 37 ± 11 years and the mean body mass index (BMI) was 21.6 ± 2.6 kg/m^2^. Mean operative time for TL and TT was 75.4 ± 27.3 and 93.5 ± 27.1 min, respectively, whereas the median operative time resulted 70 and 90 min, respectively. Final histology documented benign nodules in 323 (55.3%) cases, whereas papillary thyroid carcinomas and follicular thyroid carcinomas were reported in 252 (43.3%) and 8 (1.4%) cases, respectively. Postsurgical complications occurred in 37 cases (6.3%), among which 8 (1.4%) unconventional complications related to the access from the axilla to the thyroid. Unconventional complications included 4 seromas (0.7%), 1 track seeding (0.2%), 2 transient brachial plexus palsies (0.3%), and 1 transient Bernard–Horner syndrome (0.2%). Conversion to open surgery was required in only one case (0.2%) at the beginning of the experience due to locally advanced cancer that escaped the preoperative detection.

**Table 1:** Patient demographics, operative features, pathological findings, and surgical outcomes

Variables	Values, *n* (%); (total *n* = 583)
Gender	
Male	3 (0.5%)
Female	580 (99.5%)
Age (years)	37 ± 11
Body Mass Index (kg/m^2^)	21.6 ± 2.6
Thyroid volume (ml)	19.4 ± 10.1
Nodule diameter (mm)	25.4 ± 12.7
Operation extent	
Thyroid lobectomy	363 (62.2%)
Total thyroidectomy	220 (37.8%)
Side	
Right	331 (56.8%)
Left	252 (43.2%)
Operative time (min.)	
Thyroid lobectomy	75.4 ± 27.3
Total thyroidectomy	93.5 ± 27.1
Thyroiditis	
No	434 (74.5%)
Yes	149 (25.5%)
Histology	
Benign	323 (55.3%)
Papillary thyroid carcinoma	252 (43.3%)
Follicular thyroid carcinoma	8 (1.4%)
Hospital stay (days)	2.12 ± 0.94
Surgical complications	37 (6.3%)
Transient hypocalcemia	7 (1.2%)
Definitive hypocalcemia	2 (0.3%)
Transient RLN palsy	7 (1.2%)
Definitive RLN palsy	1 (0.1%)
Postoperative cervical hematoma	7 (1.2%)
Postoperative bleeding	3 (0.5%)
Tracheal injury	2 (0.3%)
Unconventional complications	8 (1.4%)

**Fig. 1:**
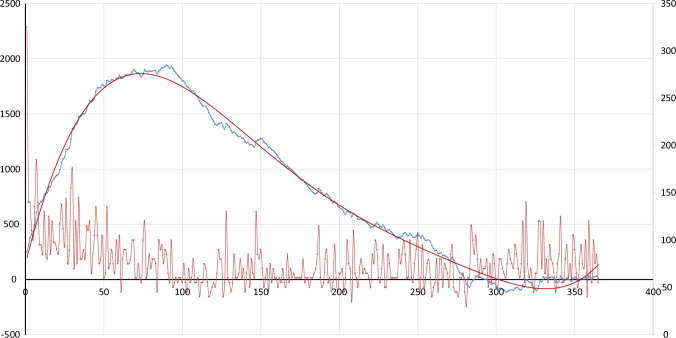
Learning curve of TL. The red line represents the operative time plotted chronologically. The blue line represents the cumulative CUSUM score. The red curve represents the CUSUM tendency extracted by a fifth-grade polynomial equation (colour figure online)

**Fig. 2:**
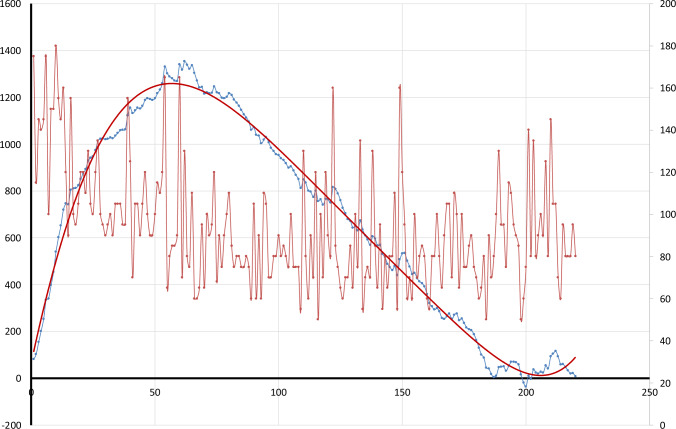
Learning curve of TT. The red line represents the operative time plotted chronologically. The blue line represents the cumulative CUSUM score. The red curve represents the CUSUM tendency extracted by a fifth-grade polynomial equation (colour figure online)

Table 2 describes the simple linear regression analysis between the operative time and the progressive number of cases stratified for the extent of surgery. A significant difference was found either for TL (*p* < 0.001) and TT (*p* < 0.001), showing a progressive improvement in terms of operative time according to the operator’s experience. Moreover, Figs. 1 and 2 showed the CUSUM analysis for TL and TT, respectively, taking into account the median operative time of the operator. For TL, the CUSUM analysis showed a turning point after case nr. 66 (Fig. 1), whereas for TT the turning point was fixed at case nr. 56 (Fig. 2). Besides, at TT nr. 56, the surgeon had already performed 71 TL.

**Table 2:** Simple linear regression analysis between "operative time" (dependent variable) and "number of cases" (independent variable) stratified for "extent of surgery"

Extent of surgery	RC	95% CI lower	95% CI upper	Pearson's *r*	*p *value
Thyroid lobectomy	− 0.032	− 0.044	− 0.021	− 0.283	< 0.001
Total thyroidectomy	− 0.043	− 0.060	− 0.025	− 0.309	< 0.001

RC: regression coefficient

Table 3 describes predictive factors for prolonged operative time, dividing cases into two groups according to the median operative time (70 min for TL and 90 min for TT). The absence of thyroiditis was found to be the only variable associated with prolonged operative time for TT (*p* = 0.044). Considering postoperative complications, no factor resulted associated with worse outcomes (Table 4).

**Table 3:** Analysis of factors influencing operative time for TL and TT

Parameter	TL operative time			TT operative time		
	< 70ʹ	≥ 70ʹ	*p* value	< 90ʹ	≥ 90ʹ	*p *value
Age (years)	38 ± 11	37 ± 11	0.216	38 ± 11	40 ± 10	0.163
BMI (kg/m^2^)	21.2 ± 2.6	21.8 ± 2.6	0.070	21.8 ± 2.7	21.9 ± 2.7	0.900
Thyroid volume (ml)	18.3 ± 9.9	18.8 ± 9.2	0.585	20.4 ± 11.8	21 ± 10.5	0.675
Nodule diameter (mm)	27.2 ± 13	28.5 ± 13	0.371	20.4 ± 10	22.7 ± 12.6	0.142
Side			0.605			0.560
Right	98 (46.9%)	111 (53.1%)		56 (45.5%)	67 (54.5%)	
Left	68 (44.2%)	86 (55.8%)		48 (49.5%)	49 (50.5%)	
Thyroiditis			0.837			0.044
No	124 (45.4%)	149 (54.6%)		70 (43.2%)	92 (56.8%)	
Yes	42 (46.7%)	48 (53.3%)		34 (58.6%)	24 (41.4%)	
Histology			0.059			0.899
Benign	107 (42.5%)	145 (57.5%)		34 (47.9%)	37 (52.1%)	
Malignant	59 (53.2%)	52 (46.8%)		70 (47%)	79 (53%)

**Table 4:** Analysis of factors influencing surgical complications (no/yes) for TL and TT

Parameter	TL surgical complications			TT surgical complications		
	No	Yes	*p* value	No	Yes	*p *value
Age (years)	37 ± 11	38 ± 11	0.863	38 ± 11	40 ± 10	0.565
BMI (kg/m^2^)	21.6 ± 2.6	21.5 ± 2.2	0.891	21.8 ± 2.6	22.8 ± 3.6	0.191
Thyroid volume (ml)	18.5 ± 9.3	20 ± 11.4	0.475	21.2 ± 11.3	16.2 ± 6.9	0.088
Nodule diameter (mm)	27.8 ± 13	30 ± 13.7	0.447	21.5 ± 11.5	23.2 ± 12	0.572
Side			0.377			0.309
Right	195 (93.3%)	14 (6.7%)		116 (94.3%)	7 (5.7%)	
Left	148 (95.5%)	7 (4.5%)		88 (90.7%)	9 (9.3%)	
Thyroiditis			0.364			0.191
No	259 (94.9%)	14 (5.1%)		148 (91.4%)	14 (8.6%)	
Yes	84 (92.3%)	7 (7.7%)		56 (96.6%)	2 (3.4%)	
Histology			0.084			0.927
Benign	241 (95.6%)	11 (4.4%)		66 (93%)	5 (7%)	
Malignant	102 (91.1%)	10 (8.9%)		138 (92.6%)	11 (7.4%)	

The trend of postsurgical complications is summarized in Table 5. We determined 4 distinct chronological phases in which patients were equally distributed (*n* Phase 1 = 146; *n* Phase 2 = 146; *n* Phase 3 = 146; *n* Phase 4 = 145). The complication’s rate did not differ between phases (*p* = 0.957).

**Table 5:** Occurrence of postoperative complications

Phase (*n* of cases)	Complications		*P* value
	No (%)	Yes (%)	
Phase 1 (1–146)	138 (94.5%)	8 (5.5%)	0.957
Phase 2 (147–292)	136 (93.2%)	10 (6.8%)	
Phase 3 (293–438)	136 (93.2%)	10 (6.8%)	
Phase 4 (439–583)	136 (93.8%)	9 (6.2%)	

## Discussion

The desire to avoid neck scarring after thyroidectomy has resulted in the development of several endoscopic and robotic remote-access techniques [23]. For a safe adoption of a novel approach, surgeons should ensure acceptable outcomes and minimize surgical complications [24]. Overall, RATT requires surgeons to become familiar with the robotic instrumentation, with flap dissection and with the new lateral view of the classic anatomy [25].

We started approaching RATT in 2012 and overall more than 800 cases have been performed at our institutions, making our case series one of the largest of the Western World. Although some reports exist regarding the learning curve of RATT, these are mostly limited to the Asian experience [17, 26, 27], with few studies from the Western World and in particular from Europe and often lacking of an accurate CUSUM analysis [15, 28, 29].

The CUSUM statistical test was developed during World War II to perform a quality control for munitions’ production. This sequential testing was used to allow the observer to decide if a production process was under control. Intuitively, new trainees may initially experience unacceptable failure rates. The learning curve is based on the turning point on whether performance ever becomes acceptable [30] and it usually refers to the number of cases after which there is a minimal variation in terms of operative time [31]. The CUSUM technique is an adequate test for the analysis of the results of new procedures, since it enables to dynamically visualize trends and patterns and provides a quantitative data of the accumulated surgical outcomes [27].

To assess the real learning curve of robotic transaxillary thyroidectomy, we performed a CUSUM analysis taking into consideration only operations performed by the surgeon who introduced this approach to our institution (GM). Overall, he performed 583 cases from 2012 to 2023, and at the beginning, he had no experience in robotic surgery. It allowed us to perform a critical analysis of the real learning curve of RATT, which is constituted by the summation of the learning curve of the surgical access, the docking of the robot, and the console time.

On the basis of our analysis, the learning curve for performing thyroid lobectomy is 66 cases, whereas for total thyroidectomy is 56 cases. The latter result is biased by the fact that before reaching 56 TT, the surgeon had already performed 71 TL which of course influenced the learning curve. These findings are in accordance to those reported by Kwak et al. for endoscopic transaxillary thyroidectomy [27], and we may conclude that the learning curve for robotic TT is 56 cases after gaining proficiency in TL.

Similarly, Lee et al. in a multicentric study [16] documented a learning curve of 40 cases for performing subtotal thyroidectomy and of 50 cases for total thyroidectomy for surgeons with endoscopic but without robotic experience. Nonetheless, the Korean experience is usually based on thin patients with a low BMI [23, 32] and, due to the Korean screening program for thyroid disease [33], operations are generally scheduled for small thyroid carcinoma [16, 17]. In particular, the mean tumor size in the case series by Lee et al. [16] was 8 mm, whereas in our case series was considerably higher (25 mm). Similarly, Kandil et al. [34] in a Western World case series with a median thyroid nodule size of 2.4 cm documented a significant persistent decrease in overall operative time after 45 cases. Notwithstanding, the same author afterward published a CUSUM analysis of the learning curve of transaxillary robotic thyroidectomy reporting two peaks, at the 69th and 134th cases [15]. The authors labeled the first peak as the point from which the learning phase was gained, whereas the second peak represents the point from which mastery of the technique had started. Moreover, the authors reported that the variability of the operative times may be related to the time taken by the surgeon to train fellows and residents [15].

Nonetheless, nowadays training programs which help young surgeons to familiarize with the robotic setup and with the peculiar lateral view of robotic transaxillary thyroidectomy are available. These training programs have been progressively refined during years and likely will allow surgeons to ascend the learning curve more quickly. This is in accordance to the paper published by Park et al. [17] which reported that the operative time for trained beginning surgeons with little or no experience in endoscopic surgery reaches the plateau after 20 less-than-total thyroidectomy.

It is important to underline that to assess the proficiency acquired in performing a procedure, the evaluation should not be limited to the operative time. We previously scrutinized the complication rate of our case series, documenting a low rate of conventional complications compared to those reported in the literature for open thyroidectomy [35–37], whereas procedure’s related complications were almost anecdotal [25]. These endearing results were confirmed even in another our previous study which reported the excellent complication’s rate of RATT even in patients beyond the American Thyroid Association statement recommendations [38, 39]. In the current study, we documented a very low rate of postsurgical complications which has remained stable during years. Similarly, Kwak et al. [27], in a CUSUM analysis of 300 patients underwent gasless endoscopic thyroidectomy, documented no evidence of turning point in the rate of transient hypocalcemia and transient vocal cord palsy.

Moreover, we performed an analysis of factors influencing either operative time or surgical complication rate. We found that total thyroidectomy in patients without thyroiditis resulted significantly longer: this little difference was already documented in another our previous study [39], although this result does not seem to present a real clinical implication. Furthermore, we did not find any predictive factor for higher rate of surgical complication.

On the other hand, in a recently published study by Park et al. [26] assessing the learning curve for single-port transaxillary robotic thyroidectomy, the authors documented that thyroiditis and the presence of lymph-node metastasis were statistically associated to longer operative time. Besides, Kandil et al. [34] reported that elevated BMI was significantly associated to longer operative time, either considering flap time or console time; nonetheless, it must be considered that the mean BMI of their cohort was 28.5 kg/m^2^ and 37% of patients were obese. Furthermore, Son et al. [40], in a retrospective study on 275 patients affected by papillary thyroid carcinoma who underwent RATT, reported that male gender, large thyroid gland, and thyroiditis significantly increased the total operative time; on contrary, there was no association between postoperative complications and clinicopathologic parameters.

This study harbors several limitations. First, the monocentric and retrospective setting of the study. Second, operative time was the only variable considered in the CUSUM analysis and may not be an accurate reflection of surgeon’s proficiency. Third, this study lacks of a comparative group which may reduce the power of the results. Fourth, the CUSUM analysis was performed only on one surgeon’s experience and, although this aspect reduces the potential heterogeneity of data, it limits the reproducibility of findings. Fifth, during years, procedures were performed with different versions (SI and XI) of the da Vinci robotic system (Intuitive Surgical, Sunnyvale Inc., CA, U.S.), which may contribute to make data heterogeneous. Finally, this study takes into consideration the learning curve of a surgeon with a huge background in minimally invasive video-assisted thyroidectomy (MIVAT) but without previous experience in robotic surgery or endoscopic transaxillary thyroidectomy; nowadays, several robotic training programs are available and, therefore, surgeons may experience a steep learning curve.

In conclusion, for a surgeon without previous robotic experience, the learning curve for performing robot transaxillary TL is 66 cases. After that, 56 cases must be performed to acquire proficiency in robotic transaxillary TT. Nonetheless, an intense training program may potentially reduce the learning curve. Moreover, although RATT has been proved safe in advanced cases, we suggest to select ideal cases at the beginning of the experience, such as women with a normal BMI and small nodule without thyroiditis, scheduled to undergo thyroid lobectomy.

## Data Availability

Data are available upon reasonable request.
